# Renal Transplant Artery Stenosis and Kinking: An Unusual Association

**DOI:** 10.24908/pocus.v8i2.16461

**Published:** 2023-11-27

**Authors:** R Haridian Sosa Barrios, V Burguera Vion, E Casillas Sagrado, D Villa Hurtado, S Jiménez Álvaro, I Martín Capón, M Fernández Lucas, Maite E Rivera Gorrín

**Affiliations:** 1 IRYCIS, Hospital Universitario Ramón y Cajal Madrid Spain; 2 Grupo de Nefrología Diagnóstica e Intervencionista de la Sociedad Española de Nefrología (GNDI) Madrid Spain; 3 UAH, Universidad de Alcalá de Henares Madrid Spain

**Keywords:** renal ultrasound, Renal Doppler, Point of Care Ultrasound(POCUS), assessment

## Abstract

Renal artery stenosis of the kidney allograft associated with kinking is not a frequent finding. As a correctable cause of graft dysfunction, it is important to diagnose it as soon as possible to avoid further graft damage and improve graft and patient survival. As pulsed wave Doppler ultrasound mapping of the graft’s renal arteries is essential to diagnose possible alterations, point of care ultrasound (POCUS) is a highly useful tool for early diagnosis. We present a case in which nephrologists performed this examination promptly allowing a timely diagnosis and treatment plan.

## Introduction

Renal artery stenosis of the kidney allograft is an infrequent finding, as is mechanical kinking of the artery. The right renal artery's greater length in comparison to the vein, limited space within the iliac fossa, and post-operative shifting in graft components all increase the likelihood of kinking. Renal artery stenosis and kinking can either coexist or kinking can result in stenosis. Nevertheless, since both these abnormalities can be corrected with timely treatment, early diagnosis is crucial to prevent permanent to the renal allograft.

Nephrologist-performed point of care ultrasound (POCUS) can be used to diagnose renal artery dysfunction in the allograft and expedite appropriate next steps in management. Doppler ultrasound mapping of the renal transplant is an effective, inexpensive, and reproducible test that provides relevant information in such scenario. We present a case in which POCUS evaluation of a renal transplant promptly identified renal artery stenosis (RAS) and led to the diagnosis of renal artery kinking.

## Case Report

**Figure 1:**
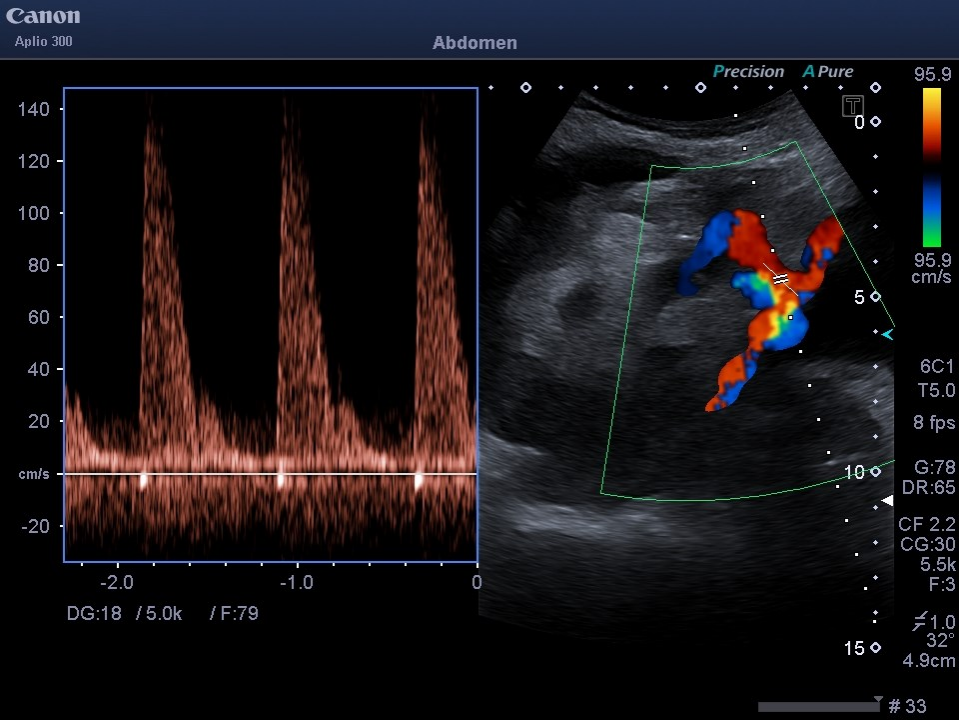
POCUS pulsed wave Doppler with aliasing at anastomosis level showing turbulent flow even when adjusted for high pulse repetition frequency to reduce noise.

**Figure 2:**
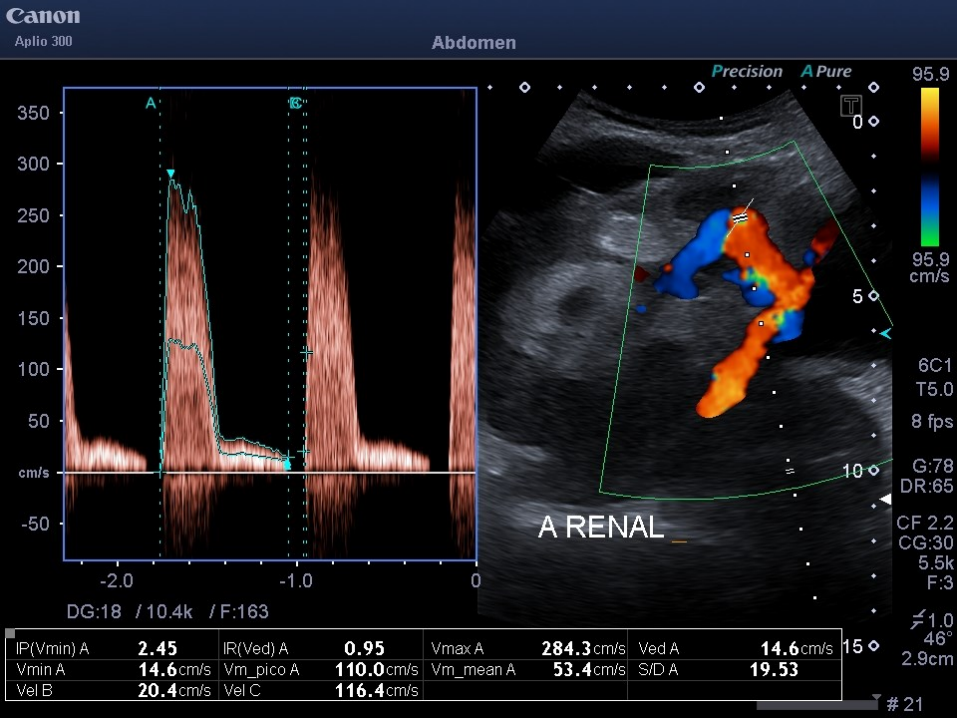
High velocities >200 cm/s at kidney transplant artery kinking. Also, aliasing at anastomosis level.

**Figure 3:**
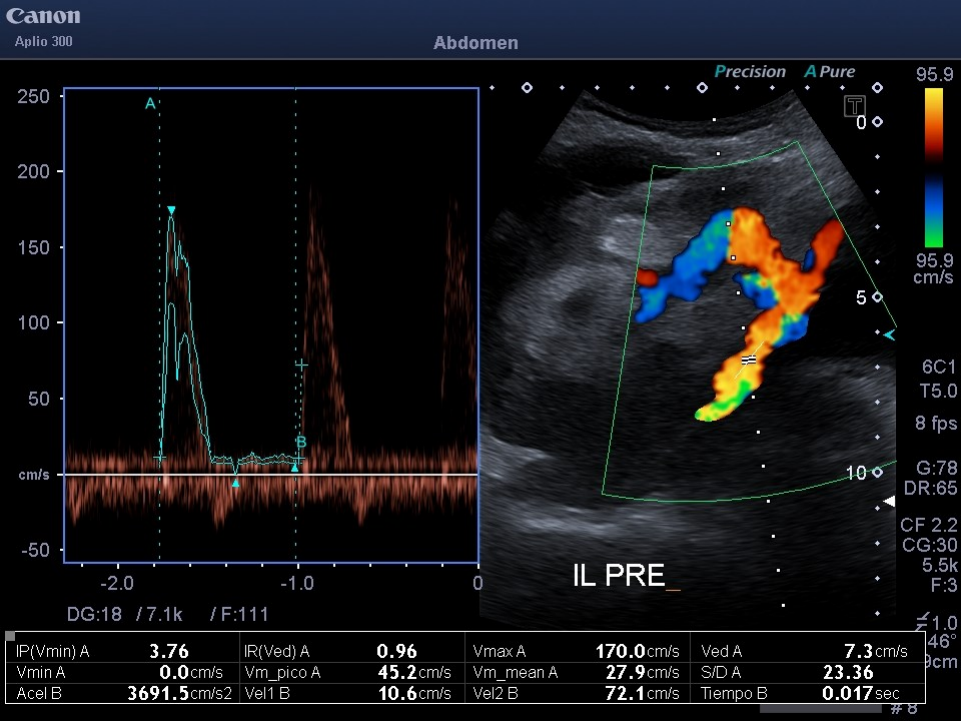
High velocities also in the iliac artery pre anastomosis.

A 61-year-old woman with end-stage kidney disease due to autosomal dominant polycystic kidney disease (ADPKD) started hemodialysis in December 2018 through a brachio-cephalic arteriovenous fistula. Her past medical history included hypertension and dyslipidemia. Eight months later, she had an expanded criteria deceased donor kidney transplant implanted in her right iliac fossa with one renal vein anastomosed end-to-side with the external iliac vein, and the renal artery anastomosed end-to-side with the external iliac artery. She had low immunological risk with 0% panel reactive antibodies and her immunosuppressive therapy included induction with basiliximab and triple therapy (tacrolimus, everolimus and steroids). A protocol ultrasound Doppler mapping of the kidney graft was done 24 hours post biopsy by an interventional nephrologist as per our center protocol, showing a normal sized graft with good general perfusion, no collections or hydronephrosis and normal intrarenal spectral Doppler registries. Serum creatinine started improving on day 6 post-transplant but halted two weeks post-operatively. Blood pressure was within acceptable levels and similar to her usual values at home (100-130/65-72 mmHg), there were no electrolyte abnormalities and cytomegalovirus viral load was undetectable. Prerenal causes were ruled out and her tacrolimus level was within goal range (7-9 ng/ml), so a kidney transplant ultrasound was performed by another interventional nephrologist, showing high velocities within the graft renal artery near the iliac artery anastomosis with aliasing (confetti-like pattern) suggesting turbulent flow, increasing in the vicinity of a kinking image near the renal hilum (Figure 1,2,3) even when pulse repetition frequency (PRF) was adjusted for high velocities (> 95.9 cm/s). The sample volume (approximately 2 mm) was placed inside the renal artery and measurements were taken at the anastomosis, along the artery and at the hilum, with an angle of insonation between 30 to 60º to minimize alterations of flow velocities and waveform blunting. These findings were not present on POCUS examination performed on postoperative day 1.

**Figure 4:**
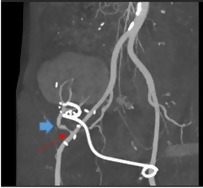
CT scan reconstruction showing both kidney transplant artery stenosis (red arrow) and kinking (blue arrow).

A computerized tomography (CT) angiogram was performed, confirming RAS at the anastomosis, and kinking of the graft renal artery (Figure 4). High velocities were also observed within the iliac artery before the anastomosis. This CT angiogram allowed radiologists to measure the diameter of the stenotic area and decide on balloon size, as well as assessing feasibility of stenting, which was deemed not suitable due to high risk of thrombosis.

After a careful multidisciplinary evaluation, a sequential approach was devised: endovascular angioplasty of the stenotic area without stenting (due to high thrombotic risk secondary to associated kinking) was performed, but unsuccessful. Doppler parameters suggestive of RAS persisted (peak systolic velocity 284.3 cm/s at 46º insonation angle) with deteriorated renal function, and open surgery vascular reconstruction was carried out a week after angioplasty: shortening of the renal artery with reimplantation to iliac artery was performed. Within a week, there was a graft function improvement and hemodynamic parameters on the follow up POCUS were within normal range.

## Discussion

Transplantation is the best renal replacement therapy nephrologists can offer, providing better survival and quality of life, generally. Improving kidney transplant outcomes remains a primary challenge from both medical and surgical point of view. RAS refers to a narrowing within the renal artery, where the lumen must exhibit a minimum reduction of 50% to hold hemodynamic significance. Renal artery Doppler US is usually the first line imaging test and universally accepted criteria for RAS diagnosis is a peak systolic velocity of >180 cm/s at the level of stenosis 1. RAS of the kidney graft is a correctable cause of hypertension and graft dysfunction in kidney transplant recipients. Its incidence is widely variable, ranging from 1 to 23% 2, and several risk factors may contribute to its development, like extended criteria donors, surgical technique, atheroma, and immunological vascular damage. Kinking of the graft artery is rare in association with stenosis, worsening prognosis as kinking renders angioplasty less effective. As mentioned, kinking of the transplant renal artery is usually related to graft malposition, lack of space in the iliac fossa and a longer graft artery with shorter vein, and its incidence is very low with few cases reported 3, 4, 5, 6.

Mapping of the renal transplant using POCUS is an inexpensive and reproducible test that provides relevant information 7. At our tertiary center, our Unit has a transplant ultrasound protocol where all patients have an examination with both two-dimensional and Doppler POCUS on day one post operatively, then every 48 hours until discharge and whenever the treating physician deems necessary. A formal report is generated, validated and images are archived for every scan.

We have a specialized Diagnostic and Interventional Nephrology (DIN) Unit which has been operating since 1991, with all medical staff trained in Doppler ultrasonography of both native (NK) and transplanted kidneys (KT). Starting in 1991, renal biopsies and 2D renal ultrasound examinations were performed by nephrologists on both NK and KT cases with an average of 2200 ultrasound scans, 500 Doppler studies and 100–120 biopsies per year with a daily scheduled list 8, 9, 10. All our Nephrology specialist registrars spend at least 6 dedicated months during the 4-year training period in the DIN Unit, excluding on call procedures.

Since 2008 our Section has accommodated approximately 20 external Nephrology specialist registrars each year from national hospitals. Additionally, we have been providing educational workshops at a national scale since 2013, focusing on kidney ultrasound, arteriovenous fistula mapping and Doppler assessment.

A structured POCUS examination should be performed in all kidney transplant patients. Complete pulsed wave Doppler mapping of the graft’s renal arteries is essential to diagnose possible alterations, as more than one issue can arise. Kinking is rare and should be kept in mind 11, 12, even if a stenosis is seen, like in this case, the pathology leading to hemodynamic changes could be other than RAS, change prognosis and management. In our case, we believe the lack of abdominal space due to ADPKD could have played a role in the renal artery kinking. 

## Conclusion

In conclusion, we believe a protocolized Doppler ultrasound mapping of the transplanted kidney is essential when graft dysfunction is observed. This case illustrates the importance of POCUS for nephrologists: early diagnosis is the key, and nephrologists with proper training in POCUS can promptly perform this examination.

## Conflict of Interest 

All authors declare there are no conflicts of interest. 

## Ethical approval

All procedures performed in studies involving human participants were in accordance with the ethical standards of the institutional research committee and with the 1964 Helsinki declaration and its later amendments or comparable ethical standards. This article does not contain any studies with animals performed by any of the authors. 

## Informed consent

Informed consent from the patient was obtained. All images were anonymized to reduce likelihood of identification. 
